# Onychoscopic Analysis of Cyclophosphamide-Induced Polydactylic Bluish-Gray Discoloration in a Case of Pemphigus Foliaceus

**DOI:** 10.7759/cureus.57233

**Published:** 2024-03-30

**Authors:** Priya Garg, Ashwini D Mundhe, Avinash Jadhav, Nishtha Mishra, Kirti S Deo

**Affiliations:** 1 Dermatology, Dr. D. Y. Patil Medical College, Hospital and Research Centre, Pune, IND

**Keywords:** pemphigus foliaceus, nail apparatus melanoma (nam), chromonychia, chemotherapeutic agents, dexamethasone-cyclophosphamide pulse

## Abstract

Cyclophosphamide, an alkylating agent, has rarely been observed to cause a bluish discoloration of nails, an occurrence that is typically underreported. We describe the case of a middle-aged male undergoing dexamethasone-cyclophosphamide pulse therapy for pemphigus foliaceus, who exhibited bluish-gray discoloration of the nails. It is crucial to differentiate this presentation from other conditions such as nail apparatus melanoma (NAM), which may manifest in a slightly different manner. We also report the onychoscopic findings observed in this case.

## Introduction

Cyclophosphamide, a chemotherapeutic drug, exerts its effects through hepatic metabolism, resulting in the formation of the active metabolite phosphoramide. This metabolite subsequently alkylates DNA, thereby impeding the process of replication [1]. In addition to systemic toxicity, cyclophosphamide can induce various mucocutaneous side effects, such as anagen effluvium, stomatitis, and hyperpigmentation affecting the skin, mucous membranes, nails, palms, soles, and teeth [2]. Cyclophosphamide, by activating matrix melanocytes, can lead to the development of blue chromonychia [3]. We report the clinical and onychoscopic findings of cyclophosphamide-induced polydactylic nail pigmentation in a 51-year-old patient with pemphigus foliaceus.

## Case presentation

**Figure 1:**
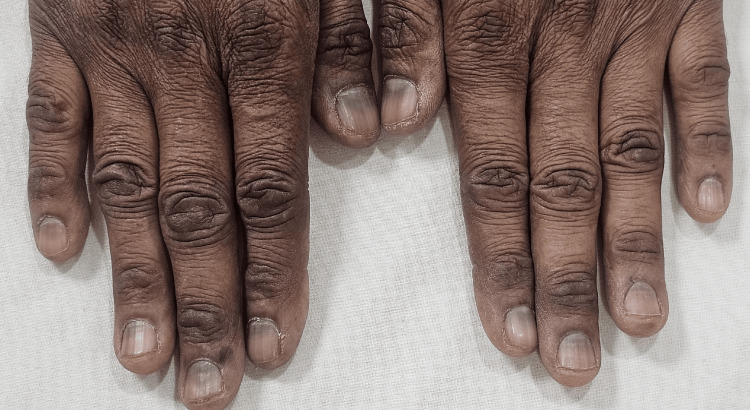
Diffuse horizontal bluish-gray discoloration on the nails of both hands.

**Figure 2:**
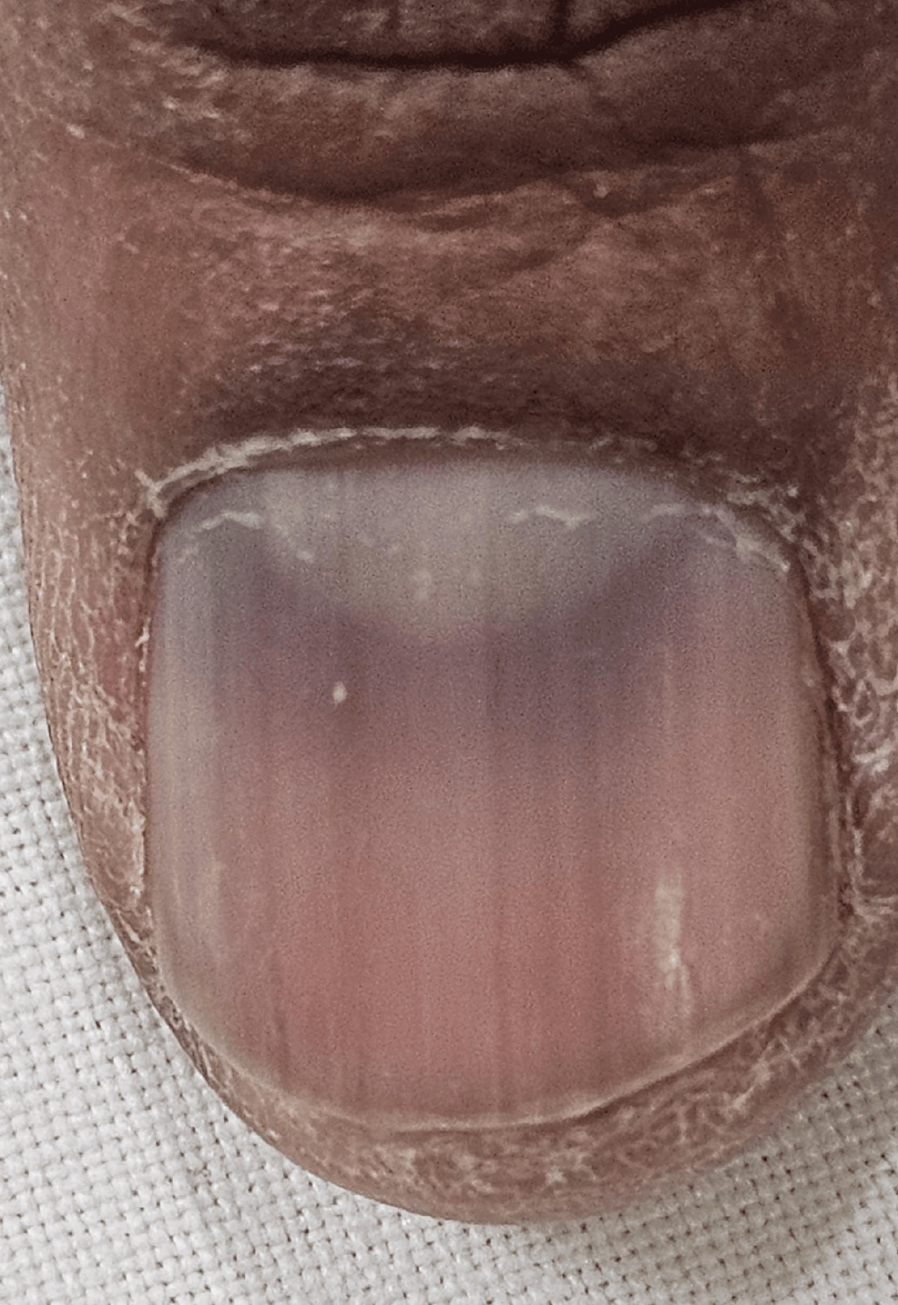
Horizontal bluish-gray discoloration seen on the thumb of the left hand.

**Figure 3:**
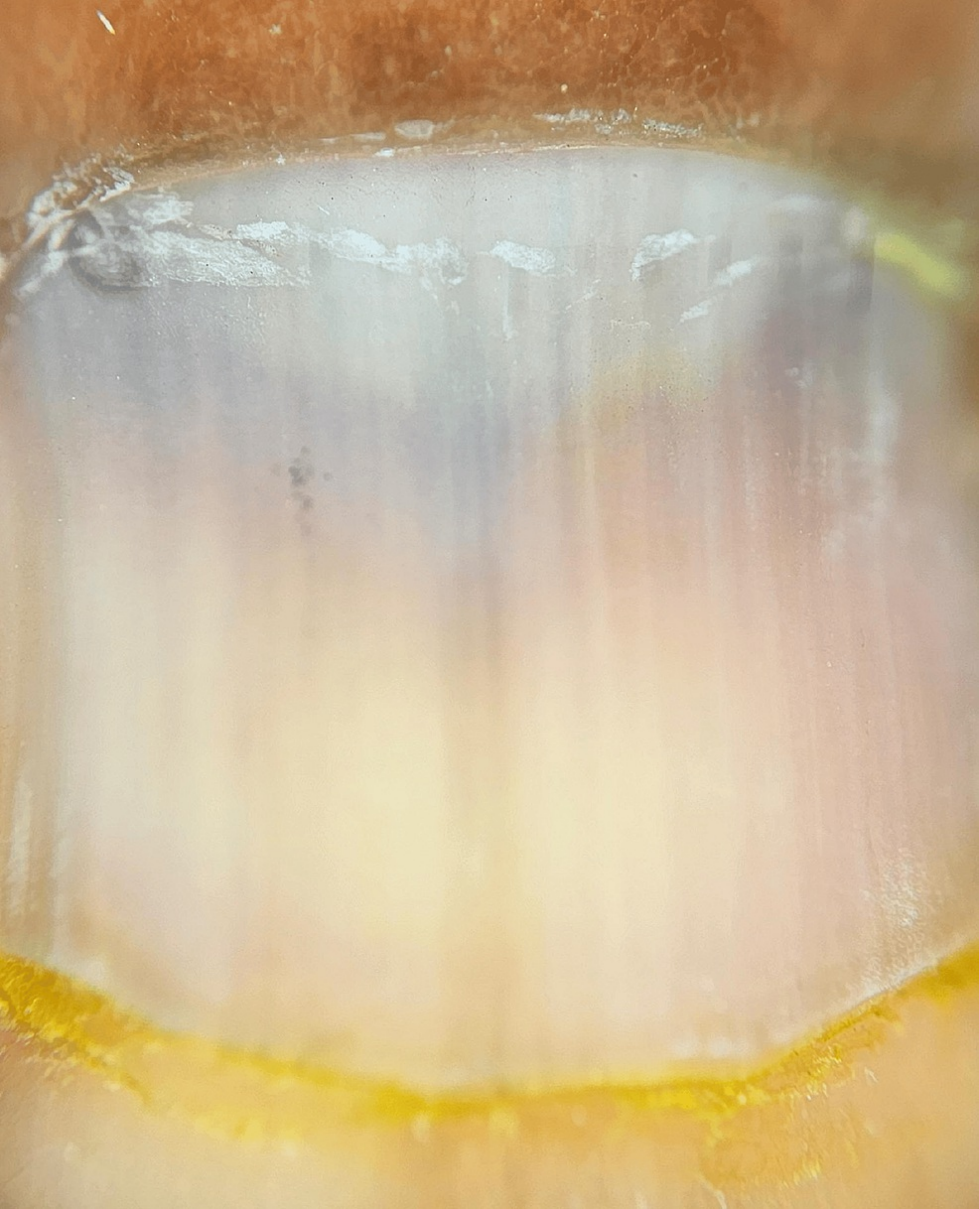
Onychoscopic examination revealed regular, parallel, uniform bluish-gray lines on a blue-gray background (Dermlite DL5).

A 51-year-old man presented with erythematous crusted plaques, erosions, and hyperpigmented patches over the V-area of the neck, back, and bilateral upper arms for 2 years. There was no other significant past or family history. The diagnosis of pemphigus foliaceus was confirmed using histopathology and immunofluorescence examination. The IgG anti-Dsg1 and anti-Dsg3 levels by ELISA demonstrated titers of 200 and 6.01, respectively. Following confirmation, the patient was started on dexamethasone cyclophosphamide pulse therapy. The treatment regimen consisted of the monthly administration of a singular intravenous dose of 500 mg cyclophosphamide, followed by a three-day intravenous infusion of 100 mg dexamethasone. Additionally, daily oral doses of 50 mg cyclophosphamide were administered during the intervals between pulses. Two months subsequent to the commencement of the treatment protocol, the patient began to develop asymptomatic, non-blanchable, diffuse horizontal bluish-gray discoloration on the nails of both hands (Figure 1). He received a cumulative dose of 4g of cyclophosphamide. The discoloration began from the proximal nail plate and progressed distally (Figure 2). Onychoscopic examination revealed regular, parallel, uniform bluish-gray lines on a blue-gray background (Figure 3). There was an absence of pigmentation in the nails of the feet, skin, and mucous membranes. Complete blood count, liver function tests (LFTs), and renal function tests (RFTs) were within the normal range. We attribute the polydactylic melanonychia to cyclophosphamide.

## Discussion

The occurrence of blue discoloration resulting from medication usage encompasses antineoplastic agents such as cyclophosphamide and doxorubicin, as well as antibiotics (minocycline), antimalarials, antiretrovirals, and anticonvulsants [4]. Drug-induced nail discoloration commonly presents with a polydactylic inclination [5]. Several mechanisms have been proposed to explain the occurrence of nail pigmentation induced by cyclophosphamide, including genetic predisposition, toxicity to the nail bed and matrix, focal activation of the melanocytic matrix, and photosensitization [6]. The pigmentation of nails, which manifests after a period of several weeks or months, exhibits a range of patterns, including diffuse, horizontal, or longitudinal streaks. It reverses several months after the cessation of drug usage [7].

It is important to distinguish this discoloration from melanonychia (brownish-black pigmentation), which may be a sign of NAM. Therefore, performing a biopsy of melanonychia is advisable to ascertain whether it is attributable to melanoma or other underlying factors [6]. The prevalence of this condition is higher among older individuals and is primarily confined to a single digit, usually the thumbnail or great toenail [4].

Therefore, it was less probable in our case. We present a noteworthy case of progressive, diffuse, bluish-gray discoloration induced by cyclophosphamide affecting all ten fingernails, accompanied by onychoscopic findings. Notably, toenails were not affected, and there was no involvement of the skin or mucosa.

## Conclusions

This particular instance highlights the importance of recognizing and differentiating drug-induced nail pigmentation from conditions like NAM, especially in those on long-term medication regimens. NAM would only affect one digit, making it highly improbable in this scenario where all 10 fingernails are involved. The onychoscopic examination was crucial in identifying the distinct features of the nail changes. Other differential diagnoses of systemic diseases that occasionally cause melanonychia, such as Addison's disease, onychotillomania, and racial melanonychia, should also be ruled out. Clinicians should remain vigilant for such adverse effects during treatment, emphasizing the need for close monitoring to ensure optimal patient care.
